# Synthesis and Biological Evaluations of Ring Substituted Tetrahydroisoquinolines (THIQs) as Anti-Breast Cancer Agents

**DOI:** 10.4172/1948-5956.1000470

**Published:** 2017-07-13

**Authors:** Suresh VK Eyunni, Madhavi Gangapuram, Bereket Mochona, Nelly Mateeva, Kinfe K Redda

**Affiliations:** 1 College of Pharmacy and Pharmaceutical Sciences, Florida A&M University, Tallahassee, USA; 2 College of Science and Technology, Florida A&M University, Tallahassee, FL-32307, USA

**Keywords:** Ring substituted tetrahydroisoquinolines (THIQs), Antiproliferative activity, Breast cancer

## Abstract

Breast cancer is a leading cause of mortality among women, resulting in more than half a million deaths worldwide every year. Although chemotherapeutic drugs remain the main stay of cancer treatment, it is observed that toxicity to normal cells poses a limitation to their therapeutic values. Moreover, the patient recovery rate from advanced breast cancer by chemotherapy is still unacceptably low. Tetrahydroisoqinoline derivatives (THIQs) were reported to act as selective subtype estrogen receptor antagonists/agonists and may serve as potential therapeutic agents for breast cancer. In continuation of previous work we systematically synthesized and characterized the tetrahydroisoquinoline (THIQs) analogs. *In-vitro* antiproliferative activity of new substituted tetrahydroisoquinoline analogs were evaluated against human ER (+) *MCF-7* (breast), ER (−) *MDA-MB-231* (breast) and Ishikawa (endometrial) cancer cell lines using the CellTiter-Glo luminescent cell viability assay. The most active compounds obtained in this study were 2b, 2i, and 3 g as demonstrated by their activity (IC_50_=0.2 μg/mL, 0.08 μg/mL; 0.61 μg/mL, 0.09 μg/mL; 0.25 μg/mL, 0.11 μg/mL) against *MCF-7* and Ishikawa cell lines respectively, in comparison to Tamoxifen activity (IC_50_=3.99 μg/mL, 7.87 μg/ml). The newly synthesized molecules were docked in the active sites of the ER-α (PDB: 3ERT), ER-β (PDB: 1QKN) and alpha-beta tubulin taxol complex (1JFF) crystal structures to determine the probable binding modes (bioactive conformations) of the active compounds.

## Introduction

Breast cancer is considered to be one of the leading causes of cancer-related deaths in women in United States. An estimated 252,710 new cases of invasive breast cancer are expected to be diagnosed in women in the USA, along with 63,410 new cases of non-invasive (*in situ*) breast cancer in the year 2017 [1]. Estrogen Receptor (ER), Progesterone Receptor (PR) and their associated steroid hormones play an important role in the development, differentiation and function of normal breast and endometrial cells. Endocrine therapy is usually offered after post-surgery and radiotherapy for breast cancer patients. In breast cancer studies, the role of ER is an endocrine therapy efficacy predictor whereas PR’s role is unknown [2]. The two subtypes of human ER, ER-α and ER-β display different tissue distribution patterns and transcriptional activities. Blocking estrogen (E2) binding to ER receptors in ER+ve breast cancer cells by employing active inhibitors of ER, stalls the growth and multiplication of cancer cells. Hence, ER-α and ER-β remain attractive targets in the treatment of breast cancer as they are over expressed in breast cancer cells. In this regard, a steroid structure based, highly Selective Estrogen Receptor Degrader (SERD) like fulvestrant, inhibit estrogen signaling process through the ER by acting as an antagonist to the estradiol binding to the ER and decreasing the ER level in breast cancer cells [3–7]. In contrast, tamoxifen (TAM), a selective ER modulator (SERM) is non-steroidal ER antagonist and often displays undesired estrogen-like agonist activity in other tissues [8–10]. Attaching various substituents on the steroidal skeletal structure of E2 resulted in a number of marketed drugs used in the treatment of hormone-dependent breast cancer [11–14]. However, steroid based anti-breast cancer agents are risk prone to other organs and display adverse side effects. TAM has been the leading drug in treating breast cancer for more than two decades and has proven to be an effective treatment for ER (+) breast cancer, particularly in the post-menopausal women [15–18]. The limitation of TAM is that, it behaves as ER antagonist in the breast tissue and as an ER agonist in other tissues with increased risk of developing endometrial cancer [19,20].

Compounds possessing tetrahydroisoquinoline (THIQ) core structure were recently reported to be effective and selective estrogen receptor modulators and have the potential of being the therapeutic agents for treating breast cancer [21,22]. Structure activity relationship studies (SAR) of ER-α selective THIQs and THIQs incorporating rigid and conformationally restricted side chains were reported by Renaud et al. [23,24]. Natural or synthesized THIQs are known to exert antiproliferative effects on cancer cells by inducing cell cycle arrest at G2/M phase [25–28]. New steroidomimetic THIQs were also reported, to act as microtubule disruptors [29]. In this regard, we also recently reported the synthesis of our most effective cytostatic THIQ compounds, which blocked replicative processes at the G2 growth phase. They proved to be effective in halting proliferation without any observed toxicity. These findings appear to suggest that some specific THIQs might work independently of the ER, e.g., by holding the microtubule network static, thereby preventing mitosis [30].

In our recent studies [31], we reported the most potent substituted THIQ 1, as the lead structure. Here we report the synthesis of new ring substituted THIQs and their *in-vitro* anti-proliferative activities were tested against *MCF-7, MDA-MB-231* human breast cancer cell lines and Ishikawa human endometrial adenocarcinoma cell lines. Tamoxifen (TAM), Raloxifene (RAL) and 4-Hydroxytamoxifen (4-OHT) were used as reference compounds. *In-silico* docking analysis and probable binding modes of these compounds were determined by mapping the active sites of the ER-α-4-OHT complex (PDB: 3ERT), ER-β-RAL complex (PDB: 1QKN), and α-β Tubulin-Taxol complex (PDB: 1JFF).

## Materials and Methods

### Experimental section

#### General

Melting points were determined on a mel-temp 3.0 melting point apparatus and are uncorrected. The structures of the final compounds were confirmed by ^1^HNMR and elemental analysis. The spectra were recorded on Varian Gemini HX 300 MHz spectrometer. All chemical shifts expressed in parts per million (δ, ppm) are reported relative to tetramethylsilane (TMS) as internal standard for solution in CDCl_3_ as a solvent unless otherwise specified. Elemental analysis of the final compounds were performed by Atlantic Microlab Inc., Norcross, GA. Flash chromatography was performed on CombiFlash (Teledyne Isco) using RediSep columns. All chemicals and solvents were purchased from Sigma-Aldrich and were used without further purification.

#### General procedure (Scheme 1) synthesis of substituted tetrahydroisoquinolinium-2,4,6-trimethyl benzene sulfonate (11a-11m)

*O*-mesitylene sulfonyl hydroxylamine (MSH) (10) was used to prepare the N-amino salt as an aminating agent [32] as previously reported [31]. To an ice-cooled solution of substituted isoquinolines (20.67 mmol) in anhydrous methylene chloride and anhydrous methanol (1:1) (60 mL) was added MSH (10) (22.74 mmol) in dry methylene chloride (10 mL) over 5 min with stirring. The reaction was stirred at 0°C for 6 h at which time ether (80 mL) was added and the suspension filtered. The precipitate was recrystallized from ethyl acetate-methanol (5:1 v/v) to give substituted isoquinolinium 2,4,6-trimethylbenzene-sulfonate salts (11a–11m) in high yields.

#### General procedure for acylation leading to ylides (12a–12m)

To an ice-cold solution of (11a–11m) (4.16 mmol) in anhydrous tetrahydrofuran (40 mL), containing triethylamine were added substituted acid chlorides (8.34 mmol). The mixture was allowed to proceed for 12 h at 70°C. After the completion of the reaction (monitored by TLC), it was quenched by adding 30 mL of saturated aqueous sodium bicarbonate solution. Extraction with dichloromethane (3 mL × 100 mL), drying over anhydrous sodium sulfate and removal of the solvent *in vacuo* gave the crude product, which was purified on CombiFlash chromatography using ethyl acetate: hexane (3:2 v/v) mixture as eluent. The resultant mono N-acylated ylides were obtained in fair to good yields.

#### General procedure for reduction yielding the substituted tetrahydroisoquinolines (2a–2m)

The Ylides (11a–11m) (5 mmol) were dissolved in absolute ethanol (20 mL) and added drop-wise to a solution of sodium borohydride (50 mmol) in absolute ethanol (25 mL) at 0°C. The reactions were allowed to proceed for 5 h to 7 h at the same. Water (35 mL) was added, and allowed to warm up to room temperature. Extraction with dichloromethane (3 mL × 50 mL), drying over anhydrous sodium sulfate and removal of the solvent *in vacuo* gave the desired products. All substituted tetrahydroisoquinolines were purified on CombiFlash using ethyl acetate: dichloromethane (2:3 v/v) as eluent to afford pure compounds (2a–2m) in fair to good yields.

#### N-(5-bromo-3,4-dihydroi soquinol in-2(1H)-yl)-4-ethylbenzamide (2a)

Yield 65%; m.p. 192°C to 193°C; **^1^**HNMR (CDCl_3_) δ (ppm): 7.65 (d, J=8.1 Hz, 2H), 7.17 (d, J=8.1 Hz, 2H), 7.09 (s, 1H, -NH, D_2_O exchange), 6.79 (d, J=8.4 Hz, 1H), 6.58 (dd, J=2.7,5.4 Hz, 1H), 6.35 (d, J=2.1, 1.8 Hz, 1H), 3.94 (s, 2H), 3.18 (t, J=5.7 Hz, 2H), 2.86 (t, J=6.0 Hz, 2H), 2.64–2.72 (q, J=7.5 Hz, 2H), 1.18 (t, J=7.5 Hz, 3H). *Anal. Calcd.* for C_18_H_19_BrN_2_O (359.26): C 60.18; H 5.33; N 7.80. Found: C 60.07; H 5.52; N 7.68.

#### 4-Ethyl-N-(7-hydroxy-3,4-dihydroisoquinolin-2(1H)-yl) benzamide (2b)

Yield 65%; m.p. 202.3°C to 203.5°C **^1^**HNMR (CDCl_3_) δ (ppm): 7.65 (d, J=8.1 Hz, 2H), 7.17 (d, J=8.1 Hz, 2H), 7.09 (s, 1H, -NH, D_2_O exchange), 6.79 (d, J=84 Hz, 1H), 6.58 (dd, J=2.7,5.4 Hz, 1H), 6.35 (d, J=2.1, 1.8 Hz, 1H), 3.94 (s, 2H), 3.18 (t, J=5.7 Hz, 2H), 2.86 (t, J=6.0 Hz, 2H), 2.64–2.72 (q, J=7.5 Hz, 2H), 1.18 (t, J=7.5 Hz, 3H). *Anal. Calcd*. for C_18_H_20_N_2_O_2_ (296.36): C 72.95; H 6.80; N 9.45. Found: C 72.75; H 6.72; N 9.48.

#### *4-tert*-Butyl-N-(7-hydroxy-3,4-dihydroisoquinolin-2(1H)-yl) benzamide (2c)

Yield 75%, m.p. 209.1°C to 211.1°C; ^1^HNMR (CDCl_3_) δ (ppm): 7.73 (d, J=8.4 Hz, 2H), 7.47 (d, J=8.1 Hz, 2H), 7.09 (s, 1H, -NH, D_2_O exchange), 6.84 (d, J=9.0 Hz, 1H), 6.66 (dd, J=2.7, 1.8 Hz, 1H), 6.43 (s, 1H), 4.02 (s, 2H), 3.27 (t, J=5.7 Hz, 2H), 2.93 (t, J=6.0 Hz, 2H), 1.33 (s, 9H). *Anal. Calcd*. for C_20_H_24_N_2_O_2_ (324.42) C 74.04; H 7.46; N 8.64. Found: C 73.82; H 7.46; N 8.46.

#### N-(7-hydroxy-3,4-dihydroisoquinolin-2(1H)-yl)-4-methoxybenzamide (2d)

Yield 65%, m.p. 192.5°C to 194.3°C; ^1^HNMR (CDCl_3_) δ (ppm): 8.12 (d, J=8.4 Hz, 2H), 3.09 (t, J=5.8 Hz, 2H), 3.42 (t, J=6.0 Hz, 2H), 3.89(s, 3H), 4.29 (s, 2H), 6.89 (s, 1H), 6.99 (dd, J=2.7, 1.8 Hz, 1H), 7.18 (d, J=9.0 Hz, 1H),7.21 (s, 1H, -NH, D_2_O exchange), 7.76 (d, J=8.1 Hz, 2H). Anal. Calcd. for C_17_H_18_N_2_O_3_ 0.095 EtOAc: C 66.57; H 5.92; N 9.13. Found: C 66.93; H 6.28; N 8.62.

#### N-(7-hydroxy-3,4-dihydroisoquinolin-2(1H)-yl)-3-methoxybenzamide (2e)

Yield 40%, m.p. 190.5°C to 192.3°C; **^1^**HNMR (CDCl_3_) δ (ppm): 7.42–7.51 (m, 3H), 7.14 (d, J=8.0 Hz*,* 1H), 7.06 (s, 1H, -NH_2_, D_2_O exchange), 6.89 (d, J=3.0 Hz*,* 1H), 6.55 (d, J=9.0 Hz, 2H), 4.21 (s, 2H), 3.82 (s, 3H), 2.84 (t, J=6.0 Hz), 2.65 (t, J=5.8 Hz, 2H). *Anal. Calcd.* for C_17_H_18_N_2_O_3_ 0.089 EtOAc (306.19): C 66.69; H 5.93; N 9.15. Found: C 66.58; H 6.33; N 8.62.

#### N-(7-hydroxy-3,4-dihydroisoquinolin-2(1H)-yl)-2-methoxybenzamide (2f)

Yield 50%, m.p. 197.3°C to 199.4°C; **^1^**HNMR (CDCl_3_) δ (ppm): 2.67 (t, J=5.7 Hz, 2H,C_4_-H), 2.89 (t, J=6.0 Hz, 2H, C_3_-H), 3.84 (s, 3H, OCH_3_ group), 4.21 (s, 2H C_1_-H), 6.58 (d, J=9.0 Hz, 2H*,*C_6_, C_8_-H), 6.91 (d, J=3.0 Hz*,* 1H,C_5_-H), 7.04 (s, 1H, -NH_2_, D_2_O exchange), 7.14–7.28 (m, 2H, C_3′,_ C_5′_-H), 7.54 (d, J=8.1 Hz*,* 2H, C_4′_, C_6′_-H). *Anal. Calcd.* for C_17_H_18_N_2_O_3_ 0.095 EtOAc (306.72): C 66.57; H 5.92; N 9.13. Found: C 66.93; H 6.28; N 8.62.

#### 2-Ethyl-N-(7-hydroxy-3,4-dihydroisoquinolin-2(1H)-yl) benzamide (2g)

Yield 49%, m.p. 215.3°C to 215.7°C;**^1^**HNMR (CDCl_3_) δ (ppm): 7.41 (d, J=8.1 Hz, 2H), 7.23–7.30 (m, 2H), 7.21 (s, 1H, -NH_2_, D_2_O exchange), 6.88 (d, J=3.0 Hz, 1H), 6.65 (d, J=3.0 Hz, 1H), 6.48 (d, J=9.0 Hz, 1H), 4.19 (s, 2H), 3.49 (t, J=5.7 Hz, 2H), 3.02 (t, J=6.0 Hz, 2H), 2.78–2.86 (q, J=7.5 Hz, 2H), 1.24 (t, J=7.5 Hz, 3H). *Anal. Calcd*. for C_18_H_20_N_2_O_2_0.105 EtOAc: C 70.74; H 6.6; N 9.17. Found: C 70.51; H 7.08; N 8.29.

#### 4-Ethyl-N-(6-hydroxy-3,4-dihydroisoquinolin-2(1H)-yl) benzamide (2h)

Yield 55%, m.p. 220.1°C to 221.6°C; **^1^**HNMR (CDCl_3_) δ (ppm): 7.62 (d, J=8.1 Hz, 2H), 7.16 (d, J=8.1 Hz, 2H), 7.09 (s, 1H, -NH, D_2_O exchange), 6.84 (d, J=8.4 Hz, 1H), 6.56 (dd, J=2.7,5.4 Hz, 1H), 6.43 (d, J=2.1, 1.8 Hz, 1H), 3.92 (s, 2H), 3.18 (t, J=5.7 Hz, 2H), 2.84 (t, J=6.0 Hz, 2H), 2.61–2.72 (q, J=7.5 Hz, 2H), 1.21 (t, J=7.5 Hz, 3H). *Anal. Calcd.* for C_18_H_20_N_2_O_2_ 0.05EtOAc (300.78): C 71.88; H 6.70; N 9.31 Found: C 72.13; H 6.79; N 9.31.

#### 4-Ethyl-N-(8-hydroxy-3,4-dihydroisoquinolin-2(1H)-yl) benzamide (2i)

Yield 63%, m.p. 218.5°C to 220.4°C; **^1^**HNMR (CDCl_3_) δ (ppm): 7.65 (d, J=8.1 Hz, 2H), 7.17 (d, J=8.1 Hz, 2H), 7.09 (s, 1H, -NH, D_2_O exchange), 6.79 (d, J=8.4 Hz, 1H), 6.58 (dd, J=2.7,5.4 Hz, 1H), 6.35 (d, J=2.1, 1.8 Hz, 1H), 3.94 (s, 2H), 3.18 (t, J=5.7 Hz, 2H), 2.86 (t, J=6.0 Hz, 2H), 2.64–2.72 (q, J=7.5 Hz, 2H), 1.18 (t, J=7.5 Hz, 3H). *Anal. Calcd.* for C_18_H_20_N_2_O_2_ (296.36): C 72.95; H 6.80; N 9.45 Found: C 72.79; H 6.69; N 9.28.

#### N,N′-(3,4-dihydroisoquinoline-2,8(1H)-diyl)bis(4-ethylbenzamide) (2j)

Yield 50%, m.p. 113.2°C to 116.4°C; **^1^**HNMR (CD_3_OD): δ 7.96 (brs, 1H), 7.81 (d, J=8.1 Hz, 2H), 7.69 (d, J=7.8Hz, 2H), 7.33 (d, J=7.5Hz, 1H), 7.25 (d, J=8.1 Hz, 2H), 7.21 (d, J=8.1 Hz, 2H), 7.17 (s, 1H, -NH, D_2_O exchange), 7.01 (d, J=7.5 Hz, 1H), 4.25 (s, 2H), 3.36 (t, J=6.0 Hz, 2H), 3.07 (t, J=5.1 Hz, 2H), 2.62–2.73 (m, 4H), 1.26 (tt, J=7.5, 7.8 Hz, 6H). *Anal. Calcd*. for C_27_H_29_N_3_O_2_ (427.54): C 75.85; H 6.84; N 9.83. Found: C 75.59; H 6.81; N 9.82.

#### N-(8-amino-3,4-dihydroi soquinolin-2(1H)-y l)-4-ethylbenzamide (2k)

Yield 45%, m.p. 223.5°C to 224.8°C; **^1^**HNMR (CD_3_OD): δ 7.75 (d, J=9.0 Hz, 2H), 7.31 (d, J=8.1 Hz, 2H), 6.93 (t, J=7.8 Hz, 1H), 6.51–6.58 (dd, J=7.5, 4.8 Hz, 2H), 4.18 (s, 2H, –NH_2_, D_2_O exchange), 3.91 (s, 2H), 3.15 (t, J=6.0 Hz, 2H), 3.02 (t, J=6.0 Hz, 2H), 2.67–2.74 (q, J=7.8, 7.5 Hz, 2H), 1.25 (t, (t, J= 7.8Hz, 3H). Anal. Calcd. for C18H21N3O 0.015 EtOAc (296.71): C 72.89; H 6.78; N 13.76 Found: C 72.87; H 7.13; N 14.16.

#### 4-Amino-N-(7-bromo-3,4-dihydroisoquinolin-2(1H)-yl) benzamide (2l)

Yield 54%, m.p. 177.8°C to 179.2°C; **^1^**HNMR (CD_3_OD): δ 7.59 (d, J=8.7 Hz, 2H), 7.26 (d, J=5.7 Hz, 2 H), 7.13 (s, 1H, -NH, D_2_O exchange), 7.06 (d, J=8.1 Hz, 1H), 6.66 (d, J=9.0Hz, 2H), 4.03 (s, 2H), 3.15 (t, J=6.0 Hz, 2H), 3.02 (t, J=6.6 Hz, 2H). *Anal. Calcd.* for C_16_H_16_BrN_3_O 0.025 EtOAc (348.44): C 55.49; H 4.66; N 12.34 Found: C 55.15; H 4.63; N 12.06

#### 4-(Ethylbenzamido)-1,2,3,4-tetrahydroisoquinolin-7-yl 4-ethylbenzoate (2m)

Yield 60%, m.p. 195.8°C to 198.2°C; **^1^**HNMR (CD_3_OD): δ 7.74 (d, J=8.1 Hz, 2H); 8.06 (d, J=8.4 Hz, 2H), 7.42 (s, 2H, -NH D_2_O exchange), 7.38 (d, J=8.1 Hz, 2H), 7.32 (d, J=8.1 Hz, 2H), 7.23 (d, J=8.1 Hz, 1H), 7.03 (dd, J=2.4, 5.7 Hz, 1H), 6.95 (d, J=5.4 Hz, 1H), 4.13 (s, 2H), 3.23 (t, J=5.4 Hz, 2H), 3.12 (t, J=5.4 Hz, 2H), 2.67–2.79 (m, 4H), 1.26 (tt, J=7.5, 7.8 Hz, 6H). *Anal. Calcd*. for C_27_H_28_N_2_O_3_ (428.52): C 5.68; H 6.59; N 6.54. Found: C 75.41; H 6.37; N 6.52.

#### General procedure (Scheme 2) synthesis of substituted tetrahydroisoquinolines (THIQs) (3a–3l)

The aminating agent, 2, 4-Dinitrophenyl hydroxylate (13) was prepared following the reported procedure [33] and was used to make the substituted isoquinoline dinitrophenoxy salts as reported [33]. The salts were obtained after adding diethyl ether to the reaction mixture after completion, filtering the resulting suspension to yield yellow solid.

#### General procedure for the synthesis of ylides

Dry THF (10 mL) was added to the substituted isoquinoline dinitrophenoxy salts and the resulting suspension was stirred at ambient temperature for 10 min. Et_3_N (2 eq) was added to the reaction mixture and stirred well. After 15 min. the substituted acid chlorides/sulfonyl chlorides (1.5 eq) were added and the reaction mixture was stirred at ambient temperature for 3 hours. Heating the reaction mixture at 70°C for 1 hr. helps in complete conversion in some reactions, but in many instances is not necessary. For isoquinolines having –NH_2_ or –OH substituents, heating resulted in the formation of major amounts of *bis*-acylated products, hence avoided. TLC (100% ethyl acetate) revealed the product formation. Reaction mixture was quenched with saturated sodium bicarbonate solution and the compounds extracted with dichloromethane (50 mL), dried over sodium sulfate, filtered and solvent evaporated. The crude compounds were used as such for the next step without further purification.

#### General procedure for the synthesis of substituted tetrahydroisoquinolines

Absolute ethanol (10 mL) was added to the Ylides prepared by the above procedure, cooled to 0°C and stirred for 15 min. Sodium borohydride (8 eq.) was added in one portion to the reaction and stirred further at 0°C for 3 h to 5 h. TLC with hexane:ethyl acetate (1:1) as eluent showed a new spot corresponding to the product. Reaction was stopped, quenched by addition of water (3 mL), ethanol evaporated, brine (20 mL) was added and extracted using dichloromethane. The organic layer collected, dried over sodium sulfate, filtered and solvent evaporated. The residue thus obtained was subjected to CombiFlash chromatography using 0% to 100% hexane:ethylacetate gradient.

#### 4-(tert-butyl)-N-(5-Methoxy-3,4-dihydroisoquinolin-2(1H)-yl) benzamide (3a)

Yield 82%; m.p. 191°C to 193°C; **^1^**HNMR (CDCl_3_) δ (ppm): 7.62 (d, J=8.7 Hz, 2H), 7.37 (d, 8.7 Hz, 1H), 7.09 (s, (br), 1H, NH), 7.07 (t, J=7.8 Hz, 1H), 6.65 (d, J=7.8 Hz, 1H), 6.58 (d, J=7.8 Hz, 1H), 4.09 (s, 2H), 3.75 (s, 3H), 3.271 (t, J=6 Hz, 2H), 2.83 (t, J=6 Hz, 2H), 1.24 (s, 9H). *Anal. Calcd*. for C_21_H_26_N_2_O_2_. 0.15 EtOAc: C 71.72, H 7.45, N 7.97; Found C 71.95, H 7.44, N 7.50.

#### 4-(tert-butyl)-N-(5-Hydroxy-3,4-dihydroisoquinolin-2(1H)-yl) benzamide (3b)

Yield 55%; m.p. 233°C; **^1^**HNMR (CDCl_3_) δ (ppm): 7.76-7.38 (dd, J=6.3, 1.5 Hz, 2H), 7.49-7.46 (m, 2H), 6.89 (m, J=7.5 Hz, 1H), 6.71 (d, J=7.5 Hz, 1H), 6.45 (d, J=7.2 Hz, 1H), 3.93 (s, 2H), 3.036 (t, J=6.3 Hz, 2H), 2.38 (t, J=6 Hz, 2H), 1.34 (s, 9H). *Anal. Calcd*. for C_20_H_24_N_2_O_2_: C 74.04, H 7.46, N 8.64; Found C 73.92, H 7.43, N 8.51.

#### N,N′-(3,4-Dihydroisoquinoline-2,5(1H)-diyl)bis(4-ethylbenzamide) (3c)

Yield 5%; m.p. 216°C to 218°C; **^1^**HNMR (CDCl_3_) δ (ppm): 7.74 (d, J=8.4 Hz, 2H), 7.65-7.60 (m, 3H), 7.26 (d, J=8.4 Hz), 7.17-7.12 (m, 3H), 6.835 (d, J=7.5 Hz, 1H), 4.09 (s, 2H), 3.23 (t, 2H), 2.82 (t, J=6 Hz, 2H), 2.67-2.57 (m, 4H), 1.22-1.14 (m, 6H).

#### N-(5-Acetamido-3,4-dihydroisoquinolin-2(1H)-yl)-4-ethylbenzamide (3d)

Yield 60%; m.p. 295°C to 297°C; **^1^**HNMR (CD_3_OD) δ (ppm): 7.73-7.62 (d, J=8.4 Hz, 2H), 7.33 (d, J=8.1 Hz, 2H), 7.26-7.14 (m, 2H), 7.0 (d, J=6.9 Hz, 1H), 4.10 (s, 2H), 3.19 (t, J=6.3 Hz, 2H), 2.95 (t, J=6 Hz, 2H), 2.7 (q, J=7.8 Hz, 2H), 2.15 (s, 3H), 1.25 (t, J=7.8 Hz, 3H). *Calcd*. for C_20_H_23_N_3_O_2_: C 71.19, H 6.87, N 12.45; Found C 71.10, H 6.97, N 11.53.

#### N-(5-(Benzyloxy)-3,4-dihydroisoquinolin-2(1H)-yl)-4-(tert-butyl) benzamide (3e)

Yield 52%; m.p. 179°C to 181°C; **^1^HNMR** (CDCl_3_) δ (ppm): 7.70 (d, J=8.7 Hz, 2H), 7.45-7.29 (m, 7H), 7.12 (t, J=7.8 Hz, 1H), 6.78 (d, J=7.8 Hz, 1H), 6.66 (d, J=7.8 Hz, 1H), 5.08 (s, 2H), 4.18 (s, 2H), 3.33 (t, J=6.3 Hz, 2H), 2.99 (t, J=6.3 Hz, 2H), 1.32 (s, 9H). *Anal. Calcd*. for C_27_H_30_N_2_O_2_: C 78.23, H 7.29, N 6.76; Found C 78.13, H 7.20, N 6.61.

#### 4-Ethyl-N-(5-hydroxy-3,4-dihydroisoquinolin-2(1H)-yl) benzamide (3f)

Yield 32%; m.p. 218°C to 220°C; **^1^**HNMR (CDCl_3_) δ (ppm): 7.75 (d, J=8.1 Hz, 2H), 7.29-7.25 (m, 2H), 6.92 (t, J=7.5 Hz, 1H), 6.72 (d, J=8.4 Hz, 1H), 6.49 (d, J=7.2 Hz), 4.00 (s, 2H), 3.12 (t, J=5.86 Hz, 2H), 2.74-2.67 (q, J=7.62 Hz, 2H), 3.5 (t, J=6 Hz, 2H), 1.26 (t, J=7.62 Hz, 3H). *Anal. Calcd*. for C_18_H_20_N_2_O_2_. 0.05 EtOAc: C 71.88, H 6.70, N 9.31; Found C 71.48, H 6.89, N 9.06.

#### 4-Ethyl-N-(7-methoxy-3,4-dihydroisoquinolin-2(1H)-yl) benzamide (3g)

Yield 55%; m.p. 197°C **^1^**HNMR (CDCl_3_) δ (ppm): 7.68 (d, J=7.5 Hz), 7.26-7.23 (m, 2H), 7.06 (d, J=8.4 Hz, 1H), 6.77-6.73 (dd, J=8.4, 2.4 Hz, 1H), 6.55 (d, J=2.4 Hz), 4.18 (s (br), 2H), 3.77 (s, 3H), 3.34 (t, J=5.7 Hz, 2H), 2.98 (t, J=6 Hz, 2H), 2.72-2.64 (q, J=7.5 Hz, 2H), 1.24 (t, J=7.8 Hz, 3H). *Anal. Calcd*. for C_29_H_23_N_2_O_2_: C 73.52, H 7.14, N 9.03; Found C 73.71, H 7.16, N 8.90.

#### Methyl-2-(4-ethylbenzamido)-1,2,3,4-tetrahydroisoquinoline-6-carboxylate (3h)

Yield 37%; m.p. 137°C to 139°C; **^1^**HNMR (CDCl_3_) δ (ppm): 7.86-7.83 (dd, J=6.4, 1.76 Hz, 2H), 7.79-7.76 (dd, J=6.74, 1.76 Hz, 2H), 7.09-6.96 (m, 5H), 6.85-6.80 (m, 2H), 3.91 (s, 3H), 3.89 (s, 3H), 3.86 (s (br), 2H), 2.75 (t, J=6.3 Hz, 2H), 2.60 (t, J=6 Hz, 2H).

#### N-(5,8-Dibromo-3,4-dihydroisoquinolin-2(1H)-yl)-4-ethylbenzamide (3i)

Yield 61%; m.p. 170°C; **^1^**HNMR (CDCl_3_) δ (ppm): 7.72 (d, J=7.8 Hz, 2H), 7.34-7.25 (m, 4H), 4.18 (s (br), 2H), 3.34, t, J=6 Hz, 2H), 3.3038 (t, J=6 Hz, 2H), 2.73-2.65 (q, J=7.8 Hz, 2H), 1.24 (t, J=7.5 Hz, 3H). *Anal. Calcd*. for C_18_H_18_Br_2_N_2_O: C 49.34, H 4.14, N 6.39; Found C 49.56, H 4.19, N 6.25.

#### 4-Ethyl-N-(5-methoxy-3,4-dihydroisoquinolin-2(1H)-yl) benzamide (3j)

Yield 52%; m.p. 163°C; **^1^**HNMR (CDCl_3_) δ (ppm): 7.68 (d, J=8.4 Hz, 2H), 7.22-7.261 (m, 2H), 7.14 (t, J=8.1 Hz, 1H), 6.72 (d, J=8.1 Hz, 1H), 6.65 (d, J=7.5 Hz), 4.2 (s (br), 2H), 3.82 (s, 3H), 3.35 (t, J=6.3 Hz, 2H), 2.92 (t, J=6.3 Hz, 2H), 2.63-2.71 (q, J=7.8 Hz, 2H), 1.23 (t, J=7.8 Hz, 3H).). *Anal. Calcd*. for C_19_H_22_N_2_O_2_. 0.05 EtOAc: C 72.49, H 7.04, N 8.90; Found C 72.73, H 6.89, N 8.20.

#### 2-(4-Ethylbenzamido)-1,2,3,4-tetrahydroisoquinoline-6-carboxylic acid (3k)

Yield 18%; m.p. 251°C to 253°C; **^1^**HNMR (CD_3_OD) δ (ppm): 7.84-7.76 (m, 4H), 7.34 (d, J=7.8 Hz, 2H, 7.21 (d, J=8.1 Hz, 1H), 4.27 (s, 2H), 3.34-3.30 (m, 2H), 3.17-3.15 (m, 2H), 2.75-2.67 (q, J=7.5 Hz, 2H), 1.25 (t, J=7.5 Hz, 3H).

#### 2-(4-Methoxyphenylsulfonamido)-1, 2, 3, 4-tetrahydroisoquinolin-5-yl-4-methoxybenzene-sulfonate (3l)

Yield 27%; m.p. 182°C to 183°C; **^1^**HNMR (CDCl_3_) δ (ppm): 7.80-7.77 (m, 2H), 7.69 (d, J=7.8 Hz), 7.380 (s, 1H, NH), 7.24 (m, 2H), 7.067 (d, J=8.7 Hz), 4.22 (s, 2H), 3.89 (s, 3H), 3.31 (t, J=6.0 Hz, 2H), 3.07(t, J=5.7 Hz, 2H), 2.71-2.63 (q, J=7.8 Hz, 2H), 1.22 (t, J=7.8 Hz, 3H). *Anal. Calcd*. for C_23_H_24_N_2_O_7_S_2_: C 54.75, H 4.79, N 5.55; Found C 54.60, H 4.80, N 5.35.

#### 4.1.8 General procedure (Scheme 3) synthesis of substituted tetrahydroisoquinolines (THIQs) (4a–4f)

The respective commercially available starting materials (tetrahydroisoquinolines) were purchased and treated with NaNO_2_ to obtain tetrahydroisoquinoline-N-Oxides followed by the reduction using Zn-Acetic acid to obtain the N-Amino tetrahydroisoquinolines. The resulting compounds were used as such for the next reaction without further purification.

#### 4.1.9 General procedure for the synthesis of substituted tetrahydroisoquinolines

To an ice-cooled solution of N-amino tetrahydroisoquinolines (0.5 mmol) in anhydrous tetrahydrofuran (5 mL) containing triethylamine (1.5 mmol) was added substituted acid chlorides (0.76 mmol). The mixture was allowed to proceed for 4 h at room temperature. After completion of the reaction (monitored by TLC), it was quenched by adding 30 mL of saturated aqueous sodium bicarbonate solution. Extraction with dichloromethane (2 mL × 35 mL), drying over anhydrous sodium sulfate and removal of the solvent *in vacuo* gave the crude product, which was purified on Combiflash chromatography using ethyl acetate: hexane (3:2 v/v) as an eluent to yield the desired products.

#### N-(7-Cyano-3,4-dihydroi soquinolin-2(1H)-y l)-2-ethylbenzamide (4a)

Yield 38%; m.p. 162°C to 167°C; **^1^**HNMR (CDCl_3_) δ (ppm): 7.45-7.17 (m 6H), 6.97 (s, 1H), 4.19 (s, 2H), 3.35-3.31 (m, 2H), 3.12, t, J=5.4 Hz, 1H), 2.85-2.77 (q, J=7.5 Hz, 2H), 1.25 (t, J=7.5 Hz, 3H).

#### N-(6,7-Dimethoxy-3,4-dihydroisoquinolin-2(1H)-yl)-4-ethylbenzamide (4b)

Yield 46%; m.p. 224°C to 225°C; **^1^**HNMR (CD_3_OD) δ (ppm): 7.89 (d, J=8.1 Hz, 2H), 7.39 (d, J=8. 4 Hz, 2H), 6.85 (s, 1H), 6.89 (s, 1H), 4.71 (s, 2H), 3.89-3.85 (m, 2H), 3.25 (t, J=6.3 Hz, 2H), 2.77-2.69 (q, J=7.5 Hz, 2H), 1.26 (t, J=7.5 Hz, 3H).

#### 4-Ethyl-N-(1-isopropyl-6,7-dimethoxy-3,4-dihydroisoquinolin-2(1H)-yl)benzamide (4c)

Yield 44%; m.p. 162°C to 163°C; **^1^**HNMR (CDCl_3_) δ (ppm): 7.58 (d, 2H, J=8.4 Hz), 7.20 (d, 2H, J=8.1 Hz), 6.16 (m, 2H), 3.86 (s, 3H), 3.84 (s, 3H), 3.69 (d, 1H, J=6.6 Hz), 3.45-3.39 (m, 1H), 3.32-3.26 (m, 1H), 2.82-2.77 (m, 2H), 2.66 (q, 2H, J=7.8 Hz), 1.97-1.91 (m, 1H), 1.27-1.18 (m, 3H), 1.13 (d, 3H, J=6.9 Hz), 0.99 (d, 3H, J=6.6 Hz).

#### N-(7-Cyano-3,4-dihydroi soquinolin-2(1H)-y l)-4-ethylbenzamide (4d)

Yield 28%; Sticky solid; **^1^**HNMR (CDCl_3_) δ (ppm): 7.69 (d, J=8.1 Hz, 2H), 7.44-7.41 (m, 2H), 7.28-7.20, m(3H), 4.20 (s, 2H), 3.34-3.31 (m, 2H), 3.09 (t, J=6.0 Hz, 2H), 2.72-2.64 (q, J=7.5 Hz, 2H), 1.23 (t, J=7.8 Hz, 3H).

#### N-(6-Chloro-3,4-dihydroi soquinolin-2(1H)-yl)-4-ethylbenzamide (4e)

Yield 42%; m.p. 205°C to 206°C; **^1^**HNMR (CDCl_3_) δ (ppm): 7.68 (d, 2H, 7.5 Hz), 7.25-7.11(m, 4H), 6.949 (1H, J=8.7 Hz), 4.16 (s, 2H), 3.30 (m, 2H), 3.02 (m, 2H), 2.68 (q, 2H, J=7.5 Hz), 1.23 (t, 3H, J=7.5 Hz).

#### Methyl-2-(4-ethylbenzamido)-6-((4-ethylbenzoyl)oxy)-1,2,3,4-tetrahydroisoquinoline-1-carboxylate (4f)

Yield 12%; m.p. 150°C to 152°C; **^1^**HNMR (CDCl_3_) δ (ppm): 8.14 (m, 3H), 7.71 (d, J=7.8 Hz, 2H), 7.46 (d, J=8.4 Hz, 1H), 7.34-7.25 (m, 3H), 7.04-7.01 (m, 2H), 5.15 (s, 1H), 3.77 (s, 3H), 3.39-3.31 (m, 2H), 2.98-2.93 (m, 2H), 2.78-2.66 (m, 4H), 1.31-1.22 (m, 6H).

### Antiproliferative activity studies

The antiproliferative activities of substituted Tetrahydro isoquinolines 2a–2m, 3a–3l and 4a–4f (a total of 31 compounds) were evaluated at the Southern Research Institute (SRI, Birmingham, Alabama, USA) according to the procedure reported previously [34]. The compounds were screened against human ER (+) *MCF-7* (breast), ER (−) *MDA-MB-231* (breast), and Ishikawa (endometrial) cancer cell lines in comparison to tamoxifen (TAM), Raloxifene (RAL) and 4-hydroxytamoxifen (4-OHT).

### Material

Human *MCF-7* and *MDA-MB-231* breast cancer cell lines were purchased from the NCI. The human Ishikawa endometrial cancer cell line was purchased from Sigma Aldrich. All three cell lines were cultured in phenol red-free RPMI-1640 (Hyclone) (500 mL) supplemented with L-glutamine-dipeptide (Hyclone) (5 mL), and 10% fetal bovine serum (Atlanta Biologicals) (50 mL).

### Method

The cell lines were cultured and treated with compounds under study including the standard TAM ranging from 0.01 nM to 100,000 nM concentration in the presence of 10 nM estradiol using the previous reported method [35]. The results expressed as IC_50_ (inhibitory concentration of 50%) were the averages of three data points for each concentration and were calculated using GraphPad Prism 4.0.

### Molecular modeling studies

#### Docking method

The crystal structures of ERα-4-OHT complex (PDB: 3ERT), ERβ-RAL complex (PDB: QKN) and Tubulin-Taxol complex (PDB: 1JFF) whose coordinates were obtained from RCSB Protein Data Bank were used as a template to dock the active THIQs of present study. The crystal structures were imported into Sybyl-X 1.3 [36] modeling suite and using structure preparation tool, Chain A (ERα-4-OHT and ERβ-RAL), Chain B (Tubulin-TA1) were extracted, hydrogen atoms were added, MMFF94s force fields and MMFF94 charges were assigned to the atoms and energy minimized. The 3D structures of the substituted THIQs along with the co-crystallized ligands, 4-Hydroxytamoxifen (OHT), Raloxifene (RAL) and Taxol (TA1) were generated by Sybyl sketch and saved as single molecular file (sdf). The conformer ensembles of all the compounds to be docked were generated using OMEGA v2.4.6, OpenEye Scientific Software [37,38] prior to docking. OMEGA ensures that low strain energy conformations were retained in the ensemble. Since the complexes in the present study have bound ligands (OHT, RAL, TA1 respectively), HYBRID v3.0.1 of OEDocking [39,40] was chosen as the appropriate docking method for our studies. The dock resolution was set to ‘High” to get the best results. The scoring function used in this process to evaluate the poses in HYBRID is HYBRID_Chemgauss4 [41]. It uses Gaussian-smoothed potentials to measure the complementary nature of ligand poses within the active site.

#### Docking studies

The X-ray structure of Ligand Binding Domain (LBD) of estrogen receptors has provided a better way to understand the ER binding site and that of β chain of Tubulin-Taxol complex to understand the Taxol binding domain. The validation of the docking poses of the bound ligands (antagonists) in individual crystal structures was done using OEdocking application, HYBRID [41] after generating the receptors using “Make Receptor version 3.0.1” module in OEdocking. The re-docking of the co-crystallized ligands has been undertaken to make sure that the bound conformations of the ligands (OHT, RAL, TA1) are reproduced by the selected docking method. Best 10 poses were retrieved and were identical with the original poses of the cognate ligands in the crystal structures with root mean square deviation (rmsd) values between them being <2 A^o^, a criterion often used for the correct bound structure prediction and validation.

## Results and Discussion

### Chemistry

**Scheme 1:**
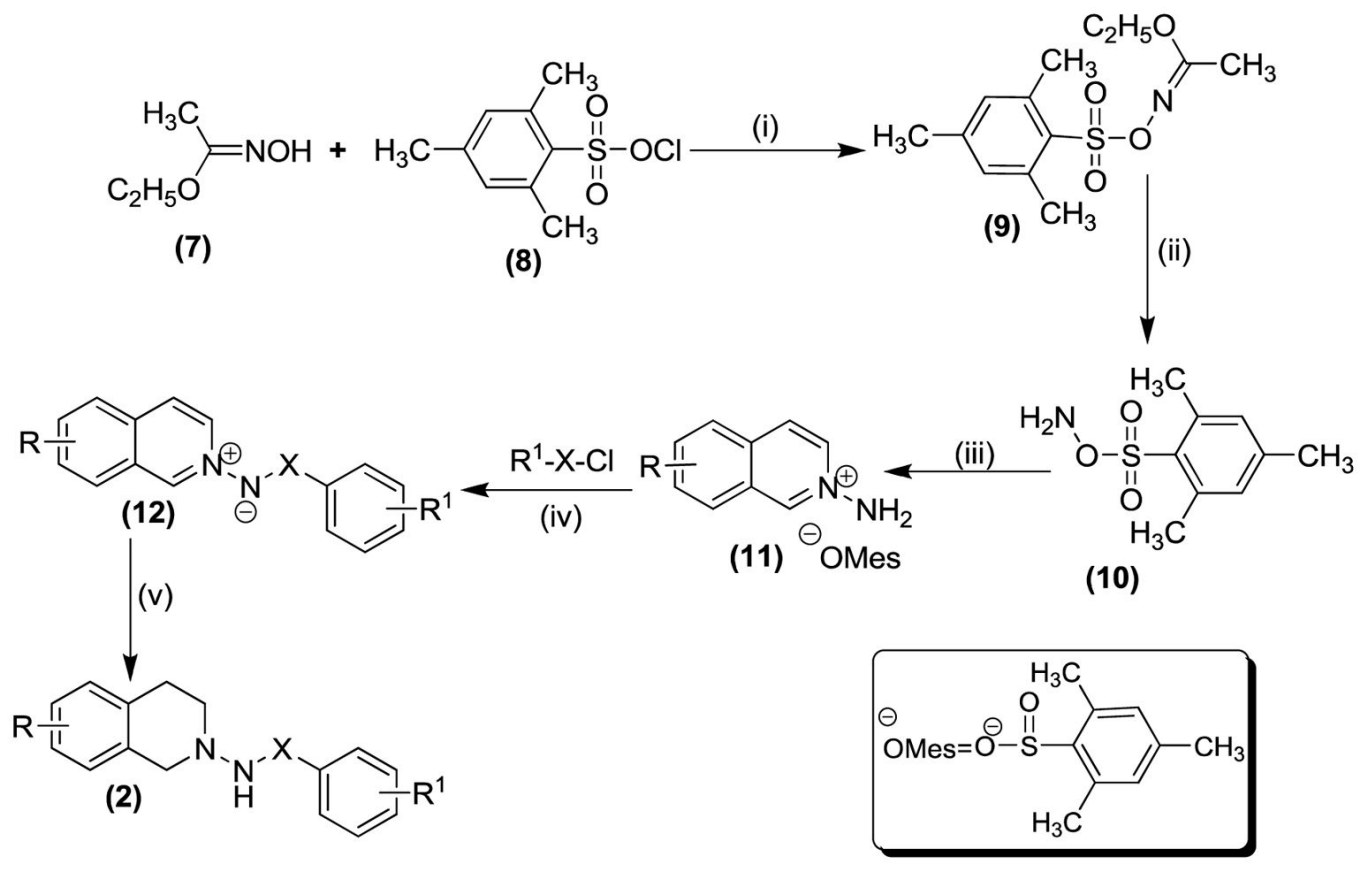
**Reaction Conditions:** (i) DMF, 0 °C, 45 min, (ii) 70 % HClO_4_, *p*-dioxane, 0 °C, 45 min, (iii) Substituted isoquinoline, dry CH_2_Cl_2_, 0 °C, 5h, (iv) 4-substituted acyl/sulfonyl chlorides, dry THF, 70 °C, 12h, (v) NaBH_4_, abs. EtOH, 7 h; R = OH, Br, NH_2_; X = CO, SO_2_; R^1^ = 4-C_2_H_5_, 3-OCH_3_, 2-OCH_3_, 2-C_2_H_5_

**Scheme 2:**
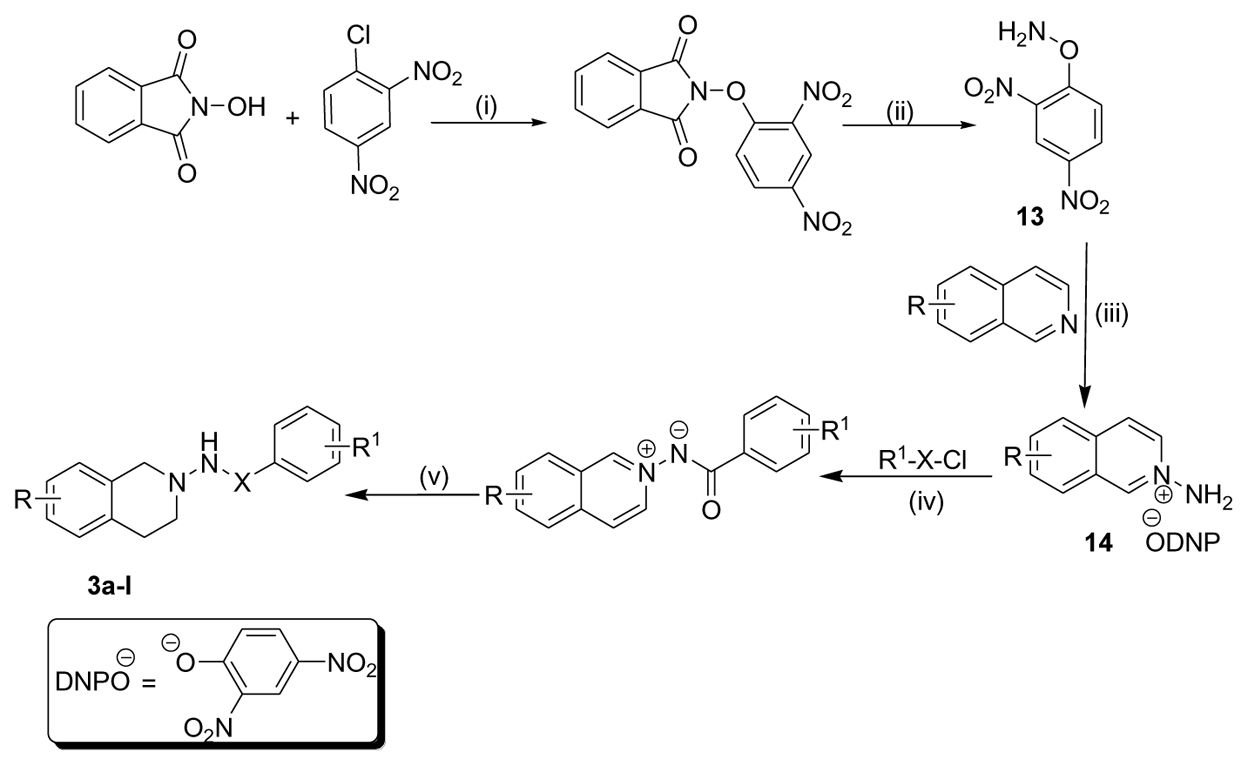
**Reaction Conditions**: (i) Et_3_N, RT, 2 hours, (ii) NH_2_.NH_2_. H_2_O, CH_2_Cl_2_, MeOH,0 °C, (iii) CH_3_CN, 50 °C,(iv) Et_3_N, THF, 25 °C, (v) NaBH_4_, EtOH, RT; X = CO, SO_2_ R= 5-OCH_3_, 5-OH, 5-NHCOCH_3_, 5-NH-CO-C_6_H_4_-C2H_5_, 5-OCH_2_-C_6_H_5_, 7-OCH_3_, 7-COOCH_3_, 5,8-di-Br, 6-COOH, 5-OSO_2_-C_6_H_4_-OCH_3_; R_1_ =4-C(CH_3)3_-C_6_H_4_, 4-C_2_H_5_-C_6_H_4_, 4-OCH_3_-C_6_H_4_

**Scheme 3:**
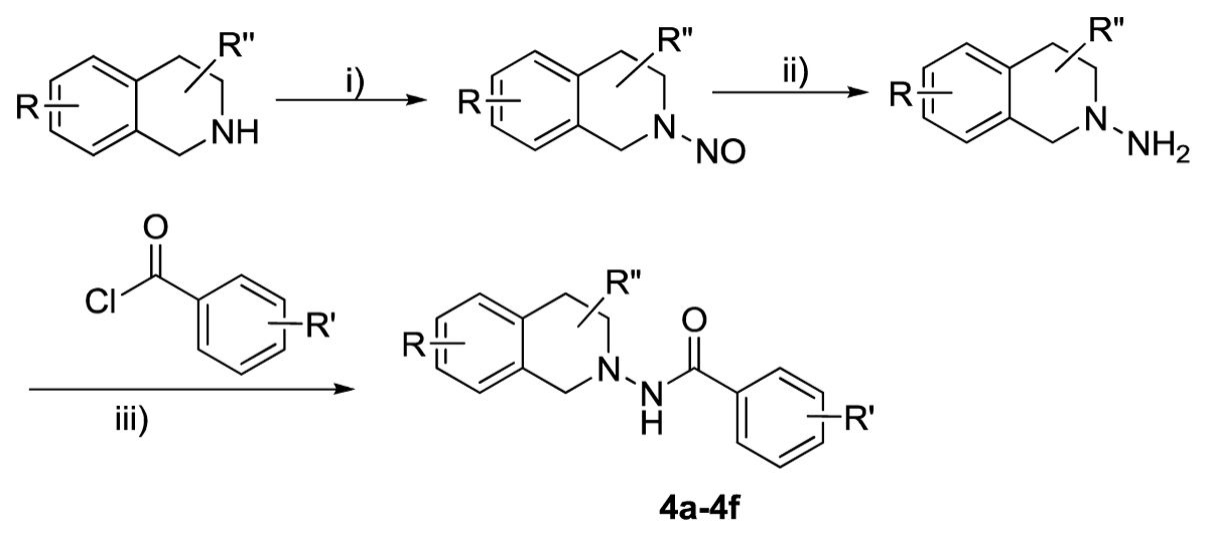
**Reaction Conditions:** i) NaNO_2_, H_2_O, 0 °C, ii) Zn, CH_3_COOH, iii) Acyl Chlorides, Et_3_N, THF; R = 7-CN, 6,7-di-OCH_3_, 6-CI, 6-OCO-C_6_H_4_-C_2_H_5_; R′ = 4-C_2_H_5_-C_6_H_4_, R″ = 1-CH(CH_3_)_2_, 1-COOCH_3_

Several substituted tetrahydroisoquinolines 2a–2m, 3a–3l and 4a–4f (a total of 31 compounds) were prepared according to three different procedures as shown in Schemes 1, 2 and 3. Compounds 2a–2m were synthesized following the Scheme 1. The aminating agent 10 was prepared using a reported procedure [32]. The amino salts of ring substituted 2-aminoisoquinolinium mesitylenesulfonates of general structure 11 used in the present study were prepared by the reaction of substituted isoquinolines and the aminating agent 10 as previously reported [31]. Reaction of 11 with corresponding substituted acylating agents (acyl chlorides) afforded N-ylides 12a–2m as stable crystalline solids. Sodium borohydride reduction of N-ylides 12a–2m in absolute ethanol furnished the title compounds 2a–2m in fair to good yields. Compounds 3a–3l were synthesized following the Scheme 2. The aminating agent 13 was prepared using a reported procedure [33]. The amino salts of ring substituted 2-aminoisoquinolinium compounds of general structure 14 were obtained with the reaction of the substituted isoquinolines and the aminating agent 13 in CH_3_CN heated at 50°C for 24 hours. Without further purification, the salts were used to prepare the N-ylides by reaction with substituted acyl/sulfonyl chlorides. Final reduction of the ylides with sodium borohydride in absolute ethanol yielded the desired THIQs 3a–3l in moderate yields. Compounds 4a–4f were synthesized following the Scheme 3. N-Oxidation of commercially available ring substituted THIQs followed by the reduction of the NO group using standard zinc/acetic acid led to the synthesis of N-amino THIQs. Acylation reaction of the isolation of N-amino THIQs with substituted acyl chlorides in the presence of base (Et_3_N) yielded the desired substituted THIQs 4a–4f in low to moderate yields.

### Antiproliferative activity

**Figure 1:**
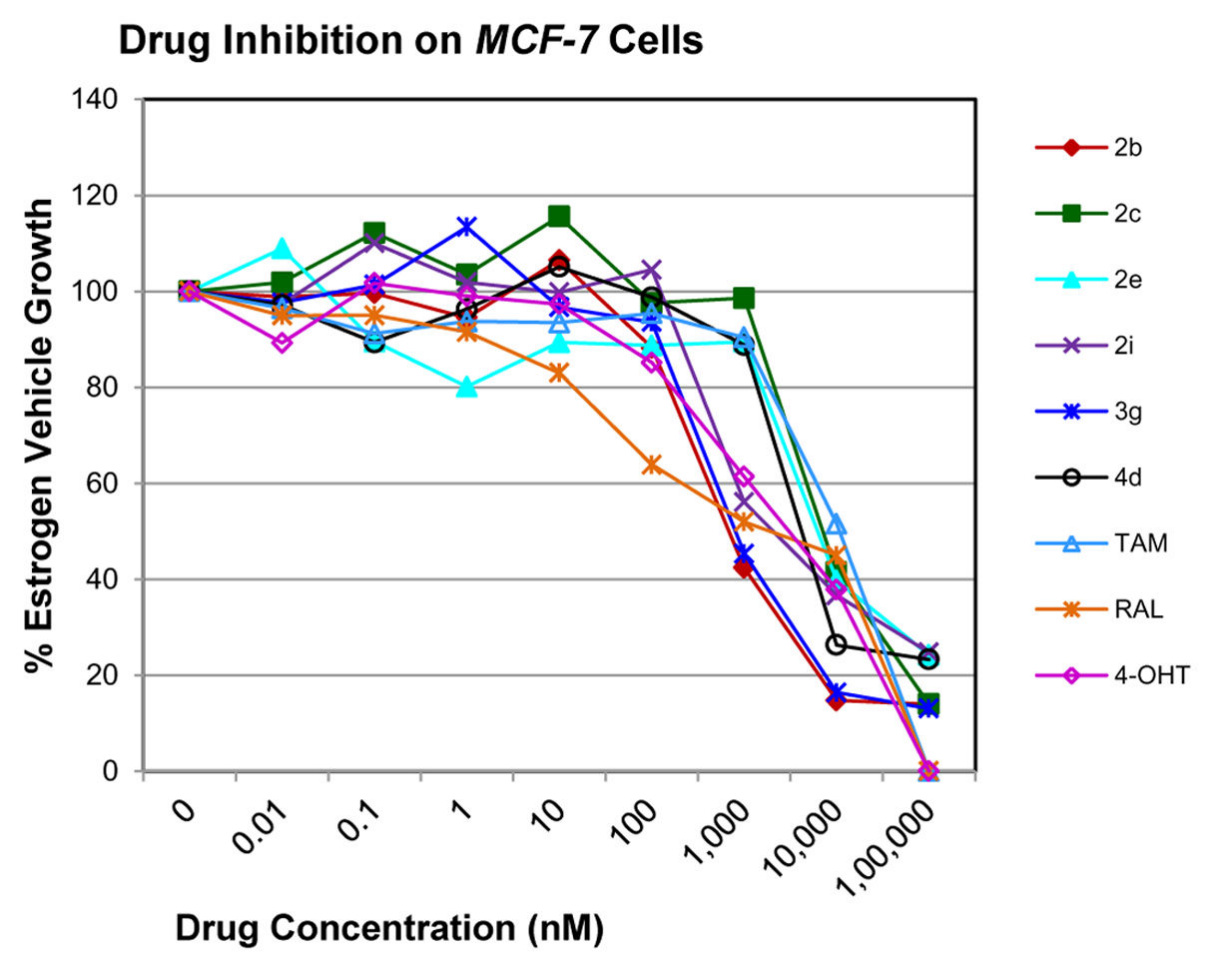
*In vitro* antiproliferative activity of selective compounds against ER (+) *MCF-7* cell line.

*In vitro* antiproliferative activity of compounds 2–4 were evaluated against human *MDA-MB-231* (ER negative breast carcinoma cell line), *MCF-7* (ER positive breast cancer cell line) and Ishikawa (endometrial) cancer cell lines at concentration ranging from 0.01 nM to 100,000 nM in the presence of 10 nM estradiol (E2) using CellTiter-Glo assay (E2 was used for competitive growth inhibitory studies). As shown in Table 1, compounds 2b, 2i, and 3g. (IC_50_=0.2 μg/mL, 0.08 μg/mL; 0.61 μg/mL, 0.09 μg/mL; 0.25 μg/mL, 0.11 μg/mL) against MCF-7 and Ishikawa cell lines, in comparison to TAM (IC_50_=3.99 μg/mL, 7.87 μg/ml). (Note: IC_50_ is the concentration of test drug where a 50% reduction is observed in cell growth compared to the untreated control after a 72 h period of exposure to test drug). This may be due to the differential affinity and binding levels of the ligand structural features to the ER receptors in the MCF-7 and Ishikawa cell lines. However, to prove the point, the expression of ER receptors by western blot is under study. Compounds bearing hydroxyl (-OH) substituents at 7^th^ position (2b) and 8^th^ position (2i) proved to be very active of the series in this cell line and exhibit higher antiproliferative activity against MCF-7 cell line based on IC_50_ value (Figure 1). However when the free hydroxyl group in compound (2b) was capped with a methyl group leading to compound (3g), the activity decreased marginally. However, the substituents at the 7^th^ position seem to be an ideal location for further manipulations of the lead compound (1). Compound (4d) with a cyano (CN) substitution at the 7^th^ position showed moderate activity (IC_50_=1.28 μg/mL). Similarly, the ethyl substitution at the para position of the aryl-acyl ring leads to better activity than other substituents in that position and other positions on the ring.

**Figure 2:**
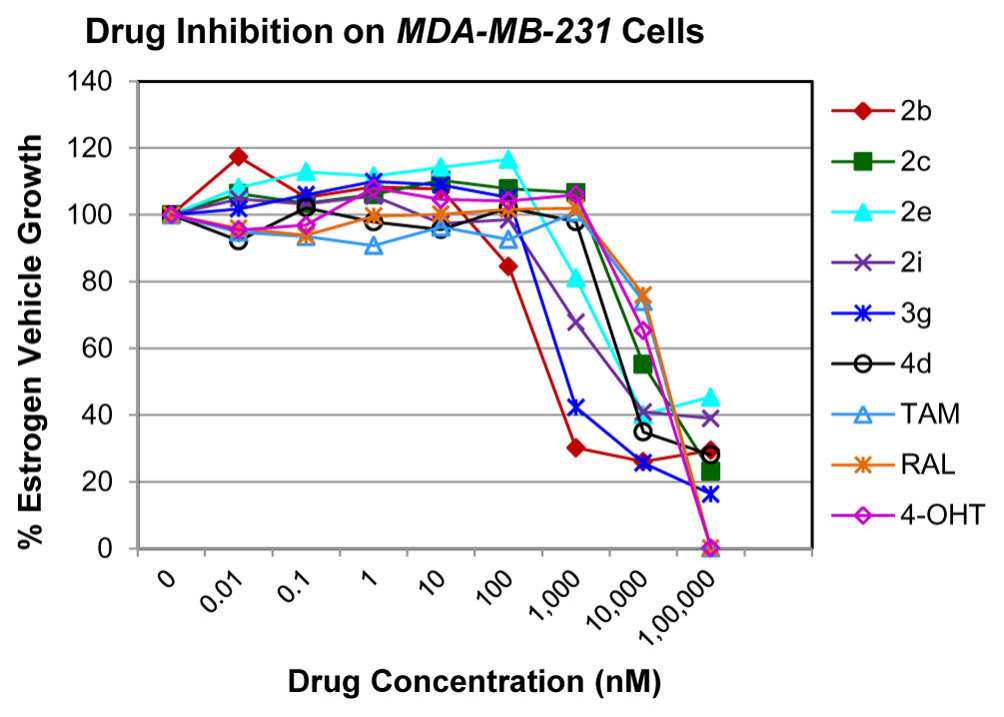
*In vitro* antiproliferative activity of selective compounds against ER (−) *MDA-MB-231* cell line.

The ER (−) MDA-MB-231 breast cancer cell line constitutes an original model for identifying the ER-independent mechanisms of TAM antiproliferative effects [42,43]. Thus, in the present study the antiproliferative activity of compounds 2–4 against human ER (−) MDA-MB-231 breast cancer cell lines were also investigated to know their mechanism of action (Figure 2). The results showed that compounds 2b, 2i and 3g (IC_50_=0.13 μg/mL, 1.36 μg/mL, 0.23 μg/mL) were more potent than TAM (IC_50_=7.85 μg/mL) shown in Table 1. Furthermore, compound 2i shows reasonable selectivity towards MCF-7 cell lines than MDA-MB-231 (IC_50_=0.61 μg/mL, 1.36 μg/mL respectively), making us believe that this particular compound may be acting as ER inhibitor. Compounds (2b) and (3g) may also inhibit cell proliferation via ER-independent mechanism in comparison to TAM. Our recently published work [31] indicates that compound (2b) acts as a potent microtubule-destabilizing agent by holding static the microtubule network, thereby preventing mitosis. Cell viability studies on similar compounds, tetrahydropyridines (THPs) made in our laboratory indicated that the dosage system used in the experiments could not lead to non-specific high toxicity effect of these compounds against normal cells. Similar studies on the tetrahydroisoquinolines (THIQs) are under investigation.

**Figure 3:**
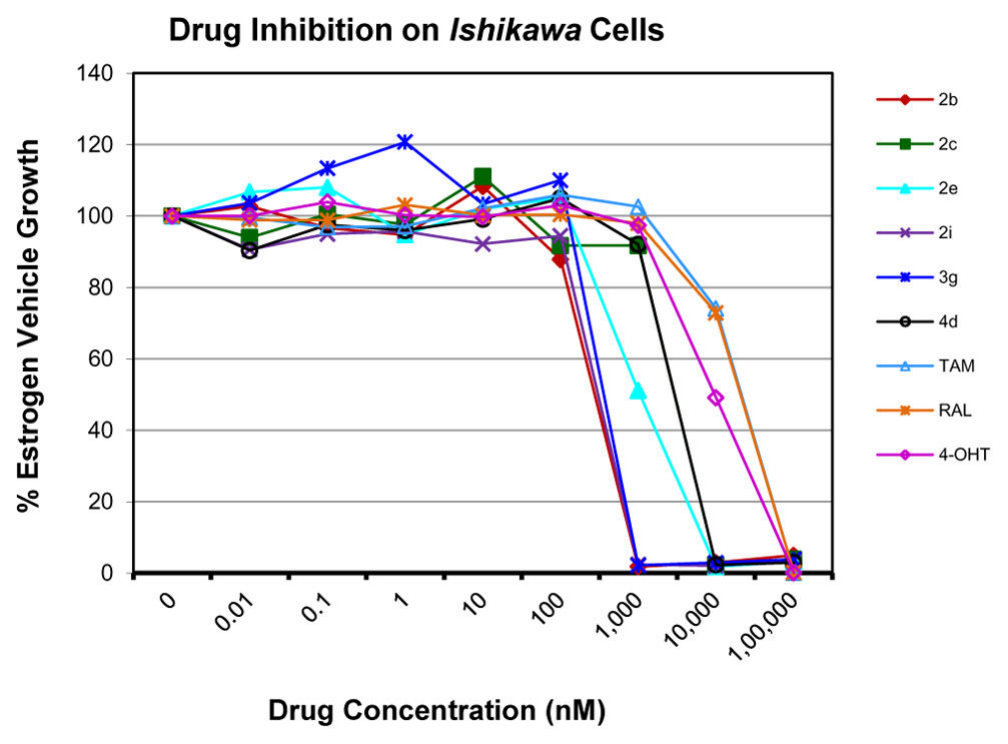
*In vitro* antiproliferative activity of selective compounds against Ishikawa cell line.

Evaluation of the antiproliferative activity of these compounds against human Ishikawa endometrial cell line were revealed that compounds 2a–2e, 2i, 2j–2m, 3g, 3i, 3j, 4d and 4e were more potent than TAM IC_50_=7.87 μg/mL (Table 1). These results indicate some of the compounds in the present study may lower the risk of developing uterine cancer based upon the IC_50_ value in comparison with TAM [35] (Figure 3).

### Molecular modeling studies

**Figure 4:**
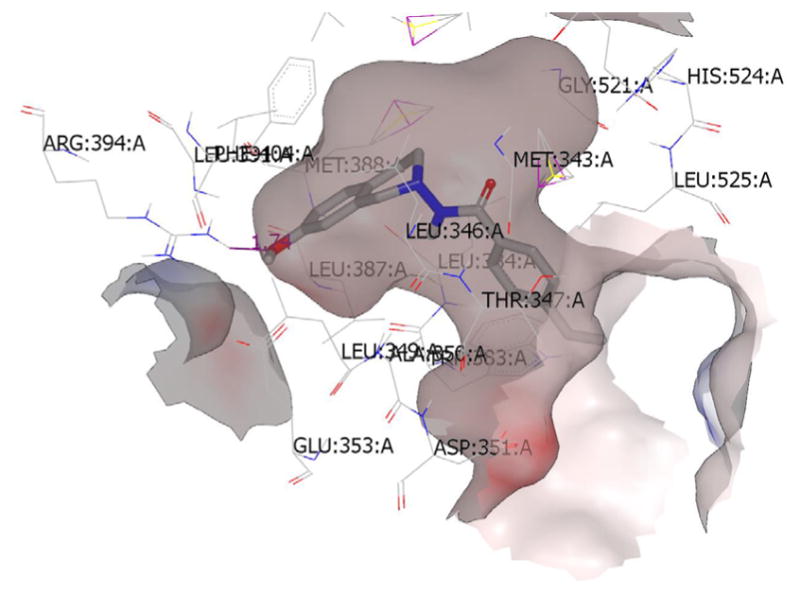
Top scoring binding pose of the most active substituted THIQ (2b) at the active site of ERα-4-OHT complex (3ERT).

**Figure 5:**
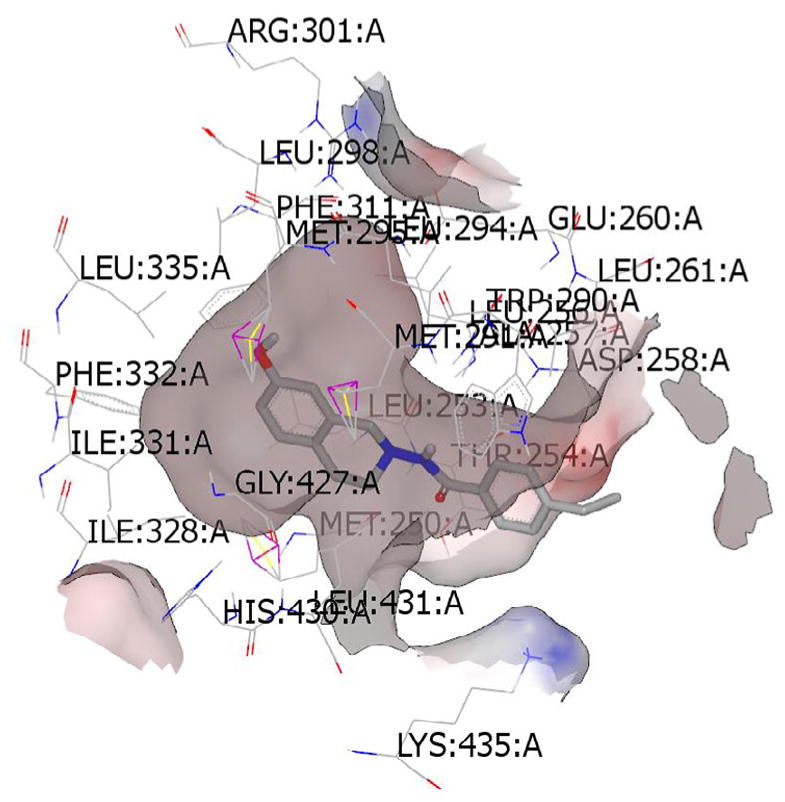
Top scoring binding pose of the most active substituted THIQ (2b) at the active site of ERβ-RAL complex (1QKN).

**Figure 6:**
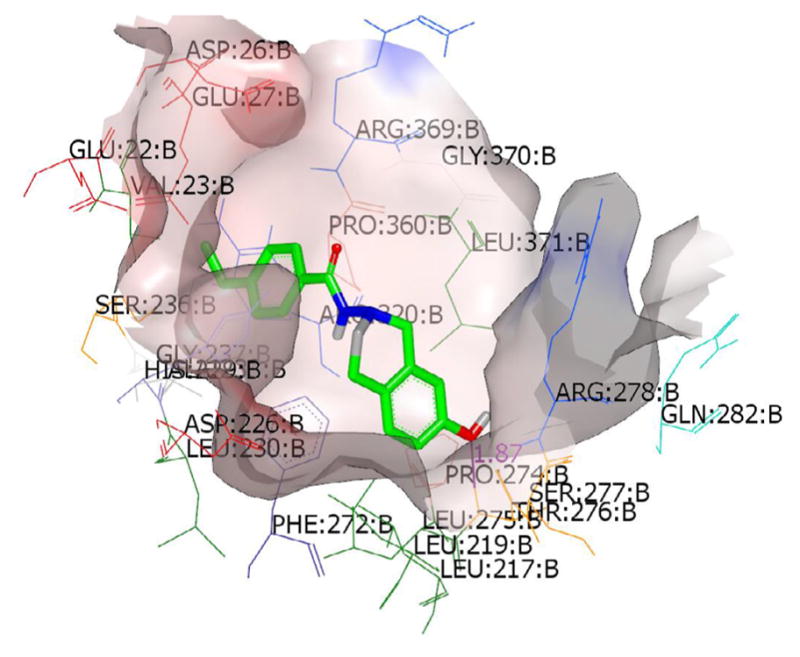
Top scoring binding pose of the most active substituted THIQ (2b) at the active site of alpha-beta tubulin taxol.

The top scoring conformations of the THIQs under present study were collected by docking the conformer ensemble (generated by OMEGA [37,38] on ERα and ERβ and Tubulin-Taxol receptors (Figures 4–6) show the preference for ERα as the ligands fit better in the bigger ligand binding pocket of ERα. These studies also give us an idea of the probable bioactive conformations and binding mode of the newly synthesized ring substituted THIQs which would assist in further optimization studies. The best compound (2b) showed hydrogen bonding interaction with Arg: 394(A) residue in the ERα-4-OHT complex. Similarly a strong hydrogen bonding with THR: 276(B) was observed for this compound in the tubulin-taxol complex. No hydrogen bonds were observed when compound (2b) was docked into the ERβ-RAL complex and the scores were relatively low compared to other molecules in the study. This may be an indication for the selectivity of the compound (2b) and other similar active compounds towards ERα and also its role as a microtubule-destabilizing activity as reported previously by us [31].

## Discussion

The antiproliferative activity of 31 new ring substituted THIQs (2, 3 and 4 series) were synthesized based on our lead structure (1) reported earlier, using three different synthetic approaches and were evaluated on *MCF-7*, *MDA-MB-231*, and Ishikawa cell lines. Their activities were compared with the reference drugs in the market, tamoxifen, 4-hydroxytamoxifen and raloxifene. In particular, the focus was on to examine the effects of the position and nature of substituents on the ring systems of lead THIQ (1). Compounds 2b and 3g with hydroxyl and methoxy substitutions on the 7^th^ position of the THIQ phenyl ring proved to be more active than the lead structure (1). The other active compound in the series was compound (2i) where the hydroxyl substitution is on the 8^th^ position of the THIQ phenyl ring. A look at the poses adapted by compound (2i) in all three docking experiments reveal that this compound scored lower than the compounds 2b and 3g. A hydrogen bonding interaction of its hydroxyl group with THR: 276(B) is observed in 1JFF but no hydrogen bonding with Arg: 394(A) was observed in 3ERT. Substitutions on the THIQ’s aliphatic six membered rings (compounds 4c and 4f) proved to be completely inactive. Simlarly, *bis* methoxy substitutions at 6^th^ and 7^th^ positions of the THIQ aromatic ring yielded inactive compounds. A comparison of the ethyl, *t*-butyl, methoxy substitutions on the acyl ring showed that the ethyl substituent at the 4-position (para) is the most optimal substitution for activity enhancement. Bis-acylated compounds 2j, 2m, 3c were less active whereas, 3d, 3l, and 4f were completely inactive. The lack of directed hydrogen bonding in the of compounds 2j, 2m, 3c, 3d, 3l, 4c and 4f might be the reason to their biological inactivity.

## Conclusion

Ring substituted THIQs based on the lead compound (1) were synthesized using three different synthetic methods in moderate yields and fully characterized. Their anti-proliferative activities against ER(+) *MCF-7* and ER(−) *MDA-MB-231* breast cancer cell lines and Ishikawa cell lines were determined and compared to that of the standard drugs in the market tamoxifen, 4-hydroxytamoxifen and raloxifene. THIQs containing hydroxyl and methoxy substituents on the 7^th^ position of the THIQ phenyl ring and ethyl substituent in the 4^th^ position of the acyl ring proved to be promising and showed more activity compared to the lead compound (1). However compound containing a hydroxyl substitution at the 8^th^ position (2i) showed significant selectivity towards ER(+) *MCF-7* breast cancer cell lines compared to ER(−) *MDA-MB-231* cell lines indicating that this particular compound may be acting via ER dependent mechanism while compounds (2b) and (3g) may be acting via ER independent mechanisms. Our recent studies have indeed showed that compound (2b) is a potent microtubule-destabilizing agent. Pose predictions and docking scores were compared for the active and inactive THIQs in the present study. The docking scores (Hybrid_Chemgauss scores) for the active THIQs were consistently higher than the inactive compounds. Within the active compounds, the scores agree with the experimental findings. Hydrogen bonding interactions between the amino acid residues of the receptors in the active site and the compounds containing OH substituents on the THQ phenyl ring were observed.

## Figures and Tables

**Table 1:** Antiproliferative activity of substituted Tetrahydroisoquinolines.

Code	Structure	IC50 μg/mL		
		Ishikawa	MCF-7	MDA-MB-231
1	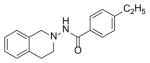	0.01	0.43	0.37
2a	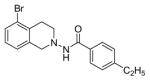	1.15	3.50	6.01
2b	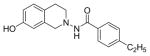	0.08	0.2	0.13
2c	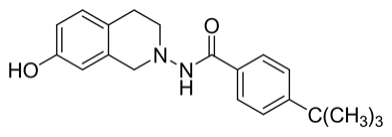	0.95	2.3	4.7
2d	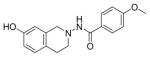	0.99	>29.83	25.17
2e	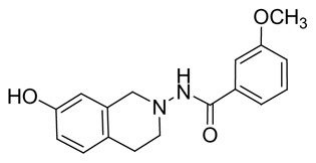	0.31	1.86	1.71
2f	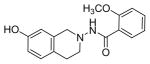	>29.83	>29.83	>29.83
2g	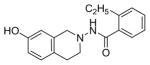	>29.64	>29.64	>29.64
2h	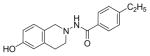	22.38	>29.64	>29.64
2i	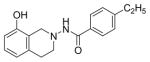	0.09	0.61	1.36
2j	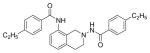	2.74	9.35	9.71
2k	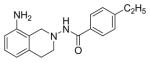	0.60	2.23	16.82
2l	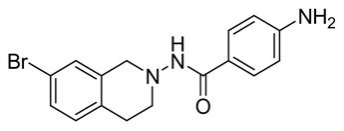	1.09	2.80	9.86
2m	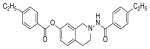	0.13	2.53	2.78
3a	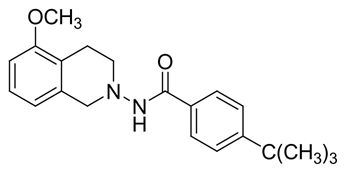	>33.84	>33.84	>33.84
3b	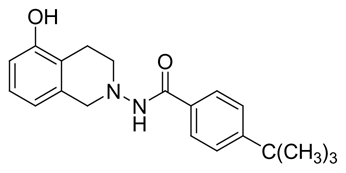	10.96	7.92	10.25
3c	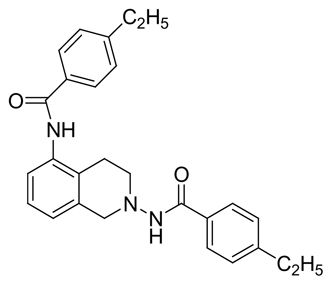	9.45	4.59	6.1
3d	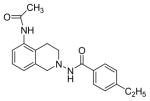	>33.74	>33.74	>33.74
3e	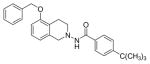	>41.45	>41.45	>41.45
3f	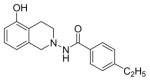	9.93	18.61	15.61
3g	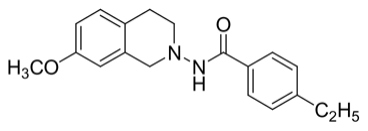	0.11	0.25	0.23
3h	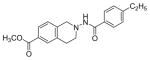	8.64	15.21	16.14
3i	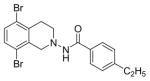	0.84	2.34	1.71
3j	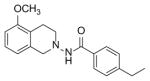	6.34	7.38	10.46
3k	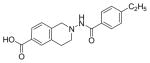	>32.44	>32.44	>32.44
3l	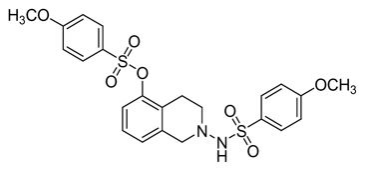	>50.46	>50.46	>50.46
4a	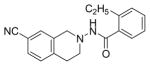	>30.54	26.45	>30.54
4b	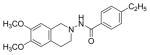	>34.10	>34.10	>34.10
4c	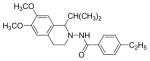	15.02	>38.25	>38.25
4d	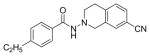	0.9	1.28	1.76
4e	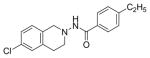	0.98	2.49	3.74
4f	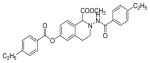	45.56	>48.65	>48.65
	Tamoxifen	7.87	3.99	7.85
	Raloxifene-Hydrochloride	10.53	0.98	11.21
	4-Hydroxy Tamoxifen	3.57	0.95	6.51
